# Are T1 values to characterize myocardial tissue equivalent between various sequences: comparison of MOLLI, shMOLLI, 3'5-MOLLI and SASHA

**DOI:** 10.1186/1532-429X-15-S1-E18

**Published:** 2013-01-30

**Authors:** Valentina O Puntmann, Tobias Voigt, Darius Dabir, Toby Rogers, Tobias Schaeffter, Eike Nagel

**Affiliations:** 1 Cardiovascular Imaging, King's College London, London, UK; 2 Medical Physics and Bioengineering, King's College London, London, UK; 3 Philips Innovative Technologies, London, London, UK

## Background

Classical modified Look-Locker sequence (MOLLI) can induce a long breath-hold and is prone to cardiac and respiratory motion. Several shorter sequences based on inversion (3'5MOLLI, shMOLLI) and saturation recovery (SASHA) of longitudinal relaxation have been proposed for derivation of T1 values or calculation of extracellular volume fraction or lambdas. Despite the validation in T1 gel phantoms, it has not been determined whether these novel sequences provide equivalent information on T1 when performed within the same individual.

## Methods

Twenty-three subjects underwent T1 mapping in a mid-ventricular equatorial short axis slice using above sequences on 3T clinical scanner prior and after gadolinium contrast (0.2 mmol/kg) administration. The images were analyzed using PRIDE tool with in-built automated motion correction. ROIs were drawn conservatively within the septal and lateral myocardium and the blood pool. Comparison of the native T1 values and lambdas were performed and expressed as percentage of mean difference from the values obtained with gold-standard MOLLI sequence.

## Results

3'5MOLLI shows the nearest approximation to MOLLI derived values with an underestimation of values in native myocardium by 1.2% and lambdas by 11%. T1 values obtained with SASHA were overestimated by 17%, and lambdas by 29%, whereas shMOLLI led to an underestimation of T1 values by 13% and lambdas by 22%.

## Conclusions

We demonstrate that 3'5'MOLLI provides near identical T1 values and its derivatives.

We propose that 3'5'MOLLI is the optimal 'shortened' sequence for clinical derivation of T1 values.

## Funding

NIHR

**Figure 1:**
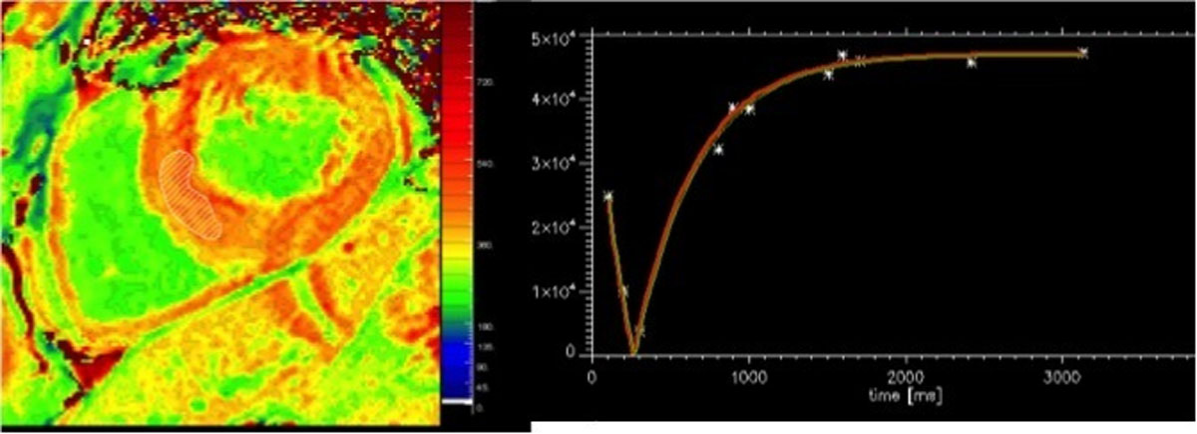
Postcontrast 3'5'MOLLI with inversion-recovery fitting curve.

**Figure 2:**
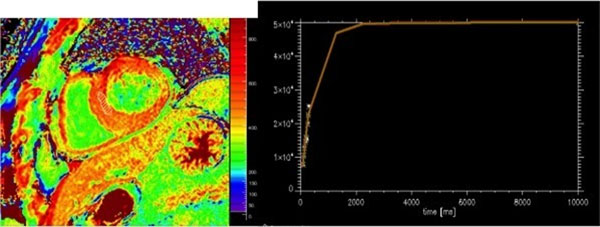
Postcontrast SASHA map with saturation recovery fitting curve.

